# Exogenous signaling repairs defective T cell signaling inside the tumor microenvironment for better immunity

**DOI:** 10.1172/jci.insight.159479

**Published:** 2022-09-08

**Authors:** Casey Moore, Joonbeom Bae, Longchao Liu, Huiyu Li, Yang-Xin Fu, Jian Qiao

**Affiliations:** 1 Department of Immunology,; 2 Department of Pathology, and; 3 Hamon Center for Therapeutic Oncology Research, University of Texas (UT) Southwestern Medical Center, Dallas, Texas, USA.

**Keywords:** Immunology, Oncology, Adaptive immunity, Cancer immunotherapy

## Abstract

It is known that tumor-reactive T cells are initially activated in the draining lymph node, but it is not well known whether and how tumor-infiltrating lymphocytes (TILs) are reactivated in the tumor microenvironment (TME). We hypothesize that defective T cell receptor (TCR) signaling and cosignals in the TME limit T cell reactivation. To address this, we designed a mesenchymal stromal cell–based delivery of local membrane-bound anti-CD3 and/or cosignals to explore their contribution to reactivate T cells inside the TME. Combined anti-CD3 and CD40L rather than CD80 led to superior antitumor efficacy compared with either alone. Mechanistically, TCR activation of preexisting CD8^+^ T cells synergized with CD40L activation of DCs inside the TME for optimum tumor control. Exogenous TCR signals could better reactivate TILs that then exited to attack distal tumors. This study supplies further evidence that TCR signaling for T cell reactivation in the TME is defective but can be rescued by proper exogenous signals.

## Introduction

Antigen-specific T cells are strongly implicated as the main driver of antitumor immunity. In humans, antigen-specific T cells are difficult to enumerate and isolate, but tumor-infiltrating lymphocyte (TIL) infiltration is correlated with better overall survival in ovarian and colorectal cancer (1, 2). Engagement of the T cell receptor/CD3 signaling complex is the first step in T cell activation in lymphoid tissues. As such, this step has been targeted by immunotherapies such as bispecific T cell engagers (BiTEs) to kill tumor cells by nonspecific TCR clustering. However, it is not well understood if and how T cells are reactivated inside the tumor microenvironment (TME), since tumor cells express low antigen/MHC (Ag/MHC) complex and lack costimulatory molecule expression. TCR clustering is not a simple on and off switch; insufficient or excessive TCR stimulation without proper costimulation can lead to T cell dysfunction or apoptosis (3–5). Furthermore, as tumors progress, TIL become unresponsive to antigens after prolonged TCR stimulation (6). During T cell priming in the lymph node, antigen-presenting cells (APCs) provide 3 signals for T cell activation: the first signal is provided by the peptide bound to MHCI (MHCI/TCR clustering), while the second is provided by costimulation (B7/CD28) and less-defined third signals, such as cytokines. It is not clear what signals are required for efficient, sustained tumor recognition and killing by TIL inside the TME. However, evidence suggests that additional interaction between DCs and T cells during reactivation in the TME may be important (7). Furthermore, it was proposed that T cells critical for response to checkpoint blockade reside in APC niches, and CD8^+^ T cell–DC cross-talk is essential for anti–PD-1 therapy in mice (8, 9). However, some studies suggest that PD-1 therapy depends on T cell activation inside lymphoid tissues rather than the TME (10).

Apart from B7 costimulation during priming, CD40 is another well-known costimulatory molecule. However, the role of each in the TME has not been well defined. CD40L is a member of the tumor necrosis factor (TNF) family normally expressed on activated CD4^+^ T cells to help license DCs for CD8^+^ T cell activation, and it is emerging as a target for antitumor immunotherapy (11–14). CD40L binding to the CD40 receptor on DCs leads to induction of costimulatory molecules and cytokine production, and it facilitates cross-presentation of antigen to CD8^+^ T cells (15). CD40 expression has also been shown to correlate with response to immunotherapy in glioblastoma and melanoma, among others (16). Ferris et al. show that CD40L plays a critical role in “DC licensing” and is required for optimal activation of CD8^+^ T cell mediated antitumor immunity (17). Furthermore, CD40 agonist therapy has efficacy in preclinical cancer models and in the clinic when used in combination with other therapies (18–20). While many studies have focused on the role of DC activation by innate stimuli and CD40 agonists systemically or in lymphoid tissues (19, 21, 22), the relative contribution of selective costimulation and TCR signal to T cell activation specifically inside the TME has not been well defined.

We hypothesize that both TCR engagement and costimulation inside the TME are defective but that exogenous TCR or certain cosignals could complement tumor-specific T cell reactivation. To test this, we designed a cell-based delivery of exogenous TCR, and costimulation (B7 versus CD40L) engagement in a membrane could form to limit their engagement to inside the TME. We expressed various forms of anti-CD3 and costimulation on the surface of mesenchymal stromal cells (MSC), which have an inflammatory tropism, and delivered them proximal to the TME. We observed that delivery of exogenous signals specifically inside the TME via anti-CD3 and CD40L combined generates superior antitumor immunity. Mechanistically, we demonstrate the indispensable and temporal role of anti-CD3 and CD40L engagement on enhancement of CD8^+^ T cell and DC interactions. Intriguingly, exogenous TCR engagement complements weak endogenous antigen presentation to rejuvenate T cells inside the TME for better immunity. Finally, locally reactivated antitumor immunity can induce systemic immunity to attack distal metastases and establish immunological memory.

## Results

### Antitumor activity of tumor-localized TCR activation and costimulation.

**Figure 1:**
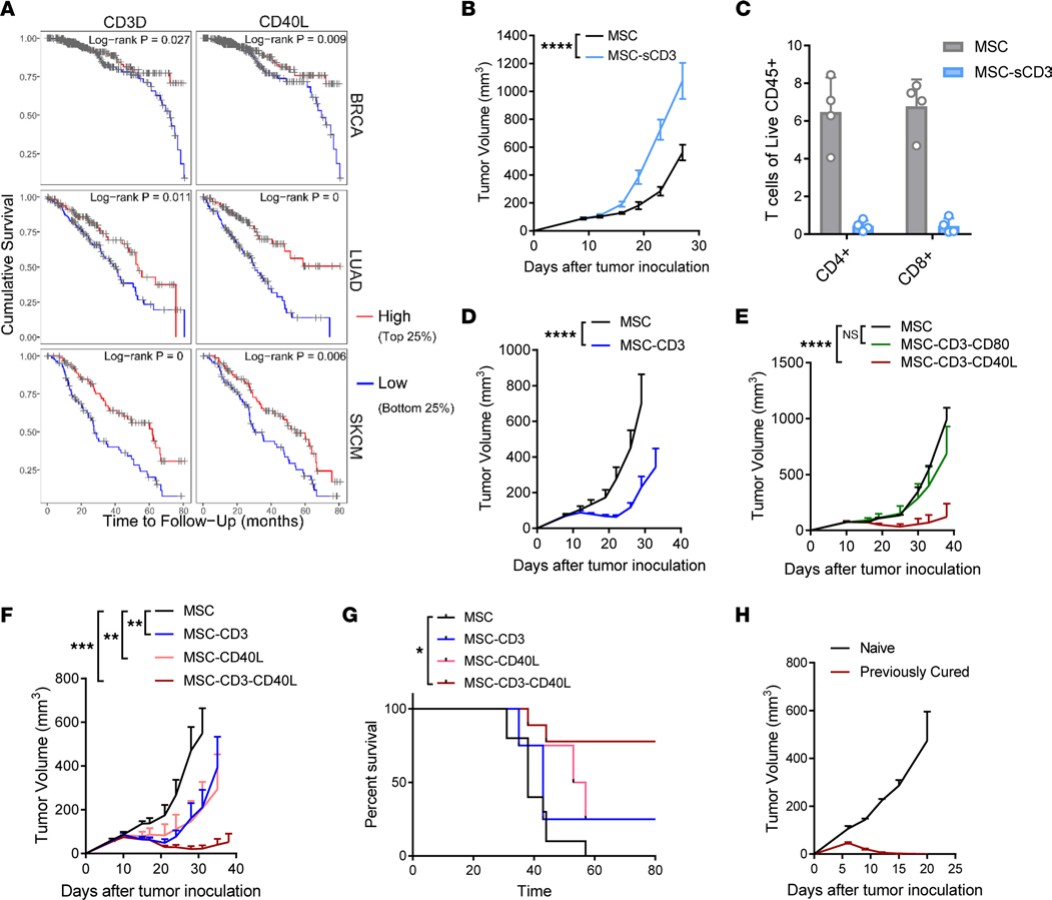
Antitumor activity of tumor-localized TCR activation and costimulation. (**A**) Cumulative survival in breast invasive carcinoma (BRCA), lung adenocarcinoma (LUAD), and skin cutaneous melanoma (SKCM) patients according to CD3 and CD40L expression (top 25% versus bottom 25%). (**B** and **C**) C57BL/6J mice were inoculated with 1 × 10^6^ MC38 tumor cells and treated peritumorally (p.t.) with 1 × 10^6^ MSC-sCD3 on day 12. Tumor growth curve (**B**) and infiltrating T cells 8 days after MSC injection (**C**) are shown. (**D**) C57BL/6J mice were inoculated with 1 × 10^6^ MC38 tumor cells and treated (p.t.) on days 11 and 13 with 1 × 10^6^ MSC or MSC-CD3 (with membrane bound anti–CD3-scFv). (**E** and **F**) C57BL/6J mice were inoculated with 1 × 10^6^ MC38 tumor cells and treated (p.t.) on days 11, 14, and 17 with 1 × 10^6^ of MSC as shown in the panel legends. (**G**) Survival curve showing percent survival over time of C57BL/6J mice treated with MSC, MSC-CD40L, MSC-CD3, or MSC-CD3-CD40L. (**H**) MC38-bearing C57BL/6J mice were treated with MSC-CD3-CD40L 3 times on days 11, 14, and 17. At 40 days after treatment, cured mice were rechallenged with 3 × 10^6^ MC38 on the opposite flank, and tumor growth was compared with naive mice. Data are presented as mean ± SEM from a representative experiment (*n* = 4 [**B** and **C**], 5 [**D**–**H**], 10 [**G**, MSC and MSC-CD3-CD40L groups], and 20 [20 cured mice from 3 independent experiments]) of 2–3 independent experiments. Statistical analysis was performed using 2-way ANOVA (**C**–**F** and **H**) or log-rank (Mantel-Cox) test (**G**). ****P ≤ 0.0001.

It is established in ovarian cancer and colorectal cancer that T cell infiltration is correlated with increased overall survival (1, 2). Similarly, we saw using the TIMER software that high CD3 expression (top 25%) is correlated with greater overall survival (23, 24) (Figure 1A). Indeed, the expression of CD3 is correlated with better prognosis in many types of cancer. This is in agreement with the notion that T cell infiltration and TCR expression are important for anticancer immunity. We hypothesized that tumor cells deliver weak antigenic signaling without proper costimulation of T cells, leading to T cell dysfunction. MC38 is a murine colon cancer with high infiltration of TILs, which allows us to study why such abundant TILs fail to limit tumor growth. If the TME fails to provide proper signaling, we want to determine if and how exogenous TCR engagement or costimulation can rescue the dysfunctional immune response inside the TME.

To test this hypothesis, we generated a system of tumor-localized delivery of TCR stimulation through engineering of mouse BM–derived MSC. MSC were harvested from mouse BM as previously described (25, 26) and transduced to express proteins of interest by lentivirus sorting on GFP or surface marker expression. We generated MSC that deliver soluble anti–CD3-Fc to activate T cells (MSC-sCD3) (Supplemental Figure 1A; supplemental material available online with this article; https://doi.org/10.1172/jci.insight.159479DS1). Surprisingly, the delivery of MSC-sCD3 to the TME led to enhanced tumor growth (Figure 1B). To explore the mechanisms for enhanced tumor growth, we looked into the number and function of TILs. When we isolated TILs, we observed a major reduction in T cells in TILs (Figure 1C). It appears that soluble CD3 can engage TCR signaling, leading to the loss of CD3^+^ cells likely through activation-induced cell death (AICD). We hypothesized that TCR ligation via anti-CD3 required membrane tethering or cross-linking, resulting in both T cell activation and expansion. Therefore, we designed an anti–CD3-scFv linked to the plasma membrane via a glycosylphosphatidylinositol (GPI) anchor (MSC-CD3), as shown in Supplemental Figure 1B. In vitro*,* the membrane-bound construct was able to induce CD25 expression on splenic CD8^+^ T cells compared with control MSC (Supplemental Figure 1C). Next, to test the in vivo efficacy of T cell receptor ligation via membrane-bound anti-CD3, we treated s.c. MC38 tumors by injecting tumor-adjacent (peritumor [p.t.]) MSC-CD3 (Figure 1D). It appears that membrane CD3 allows initial T cell activation and expansion, leading to a significant antitumor efficacy. However, tumors eventually relapsed after initial control.

We wondered whether exogenous costimulatory signals are also required to prevent T cell apoptosis or expand tumor-reactive T cells. Costimulatory molecule expression (CD80 and CD40L) in the tumor is significantly correlated with overall survival (Figure 1A and Supplemental Figure 1D). Also, we conducted single-cell RNA-Seq analysis of syngeneic mouse colon cancer (MC38) over time and found that, as tumors progress, there is less CD40L expression on CD4^+^ T cells in the TME, suggesting that this signaling may be limited (Supplemental Figure 1E). Given the improved survival in patients expressing higher levels of costimulatory molecules, we coexpressed CD80 or CD40L on MSC-CD3, generating MSC-CD3-CD80 and MSC-CD3-CD40L. To test the role of CD40L versus CD80 signaling during TCR stimulation, we treated MC38 tumors with MSC expressing anti–CD3-scFv and CD40L or CD80 compared with control MSC. While CD80 has been considered to be a leading costimulator for T cells through CD28, to our surprise, CD80 costimulation did not improve tumor control (Figure 1E). However, costimulation with CD40L in the TME led to significantly improved tumor control (Figure 1E).

To further characterize the differential effects and temporal timing of CD3 ligation and CD40L costimulation, we treated MC38 tumor–bearing mice with control MSC, MSC-CD3, MSC-CD40L, or MSC-CD3-CD40L. Both MSC-CD3 and MSC-CD40L controlled tumor growth better than MSC, but combination treatment with MSC-CD3-CD40L had the greatest antitumor efficacy and significantly increased survival (Figure 1, F and G).

To study if exogenous signals can provide systemic memory, cured mice were rechallenged with a high dose of MC38 on the opposing flank. All cured MSC-CD3-CD40L–treated mice rejected a rechallenge of MC38 (Figure 1H). This treatment is not limited to MC38. We tested its efficacy in mice bearing established CT26, which has fewer TILs. MSC-CD3-CD40L also shows antitumor efficacy in the CT26 model (Supplemental Figure 2A) and B16-OVA model (Supplemental Figure 2B), and it also leads to immunological memory in cured CT26-bearing mice (Supplemental Figure 2C). Together, the data suggest that the TME provides weak first and second signals for T cell activation, but these signals can be delivered exogenously to rescue the dysfunctional T cells.

### Antitumor effect of MSC-CD3-CD40L requires CD8^+^ T cells and DCs.

**Figure 2:**
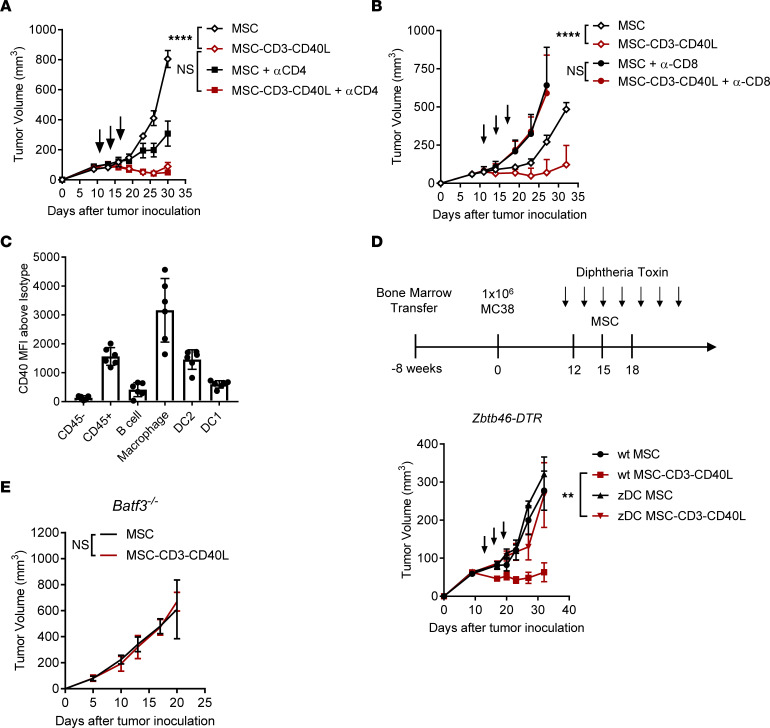
Antitumor effect of MSC-CD3-CD40L requires CD8^+^ T cells and DCs. (**A** and **B**) C57BL/6J mice were inoculated with 1 × 10^6^ MC38 tumor cells and treated peritumorally (p.t.) with 1 × 10^6^ MSC or MSC-CD3-CD40L on days 11, 14, and 17 and treated with α-CD4 (**A**) or α-CD8 (**B**) depleting antibodies twice per week beginning 1 day before treatment. (**C**) CD40 receptor MFI increase above isotype control for each subset of cells analyzed from day 11 MC38 tumor–bearing C57BL/6J mice. (**D**) C57BL/6J mice were irradiated with 2 × 550 Rad 3 hours apart and given 2 × 10^6^ to 3 × 10^6^ WT or zDC-DTR BM within 24 hours. After 8 weeks of BM reconstitution, mice were inoculated with 1 × 10^6^ MC38 tumor cells and treated (p.t.) on days 11, 14, and 17 with 1 × 10^6^ MSC or MSC-CD3-CD40L. In total, 400 ng/mouse of DT was administered i.p. every 2 days beginning 1 day before treatment. (**E**) MC38-bearing Batf3^–/–^ C57BL/6J mice were treated with MSC-CD3-CD40L 3 times on days 11, 14, and 17, and tumor growth curve is shown. Data are presented as mean ± SEM from a representative experiment (*n* = 5) of 2 independent experiments. **C** shows 2 independent pooled experiments. Statistical analysis was performed using 2-way ANOVA (**A**, **B**, **D**, and **E**). ****P ≤ 0.0001, **P ≤ 0.01.

To dissect the cellular mechanism of antitumor activity of combined TCR ligation with CD40L treatment, we began by asking which subset of T cells was most important. Antibody depletion of CD4^+^ T cells did not decrease the efficacy of MSC-CD3-CD40L therapy (Figure 2A), whereas depletion of CD8^+^ T cells led to a complete loss of tumor control in both MC38 (Figure 2B) and CT26 (Supplemental Figure 2A). This raises the possibility that CD3 and CD40L may directly engage CD8^+^ T cells for optimal activation and expansion, but we cannot rule out indirect activation. To dissect out which cells are directly responding to MSC-CD40L treatment, we analyzed the CD40 expression level on cells within the TME. Using flow cytometry, we saw that DCs, macrophages, and B cells express the CD40 receptor (Figure 2C). Therefore, it is possible that activation of these cells by CD40L on MSC might be required.

We next sought to determine which of these cell types can be activated by CD40L and contribute to tumor growth control. We used anti-CSF-1Rβ antibody treatment to deplete macrophages beginning 1 day prior to the first MSC injection, and we saw no significant change in tumor growth after treatment (Supplemental Figure 3A). We next grew MC38 tumors in *MuMt^–^* transgenic mice, which lack mature B cells, and saw that these tumors also responded to MSC-CD3-CD40L therapy (Supplemental Figure 3B). To analyze the role of DCs, we generated zbtb46-DTR BM chimeras into WT mice. This model leads to expression of the diphtheria toxin receptor (DTR) behind the zbtb46 promoter in all BM-derived cells, leading to DTR expression on the surface of classical DCs. We depleted DTR^+^ DCs 1 day before MSC-CD3-CD40L treatment to allow normal development of an antitumor immune response (Figure 2D). DC depletion by DT significantly decreased the antitumor effect of MSC-CD3-CD40L therapy (Figure 2D). To determine if a subset of DCs is essential, we used *Baft3*-deficient mice, which lack cDC1 (cross-presenting) DCs. MSC-CD3-CD40L have no antitumor effect in *Batf3^–/–^* mice (Figure 2E). This confirms that the cDC1 lineage is required for MSC-CD3-CD40L–mediated antitumor effects.

### CD40 receptor activation on DCs and CD3 ligation on antigen-specific T cells synergizes for IFN-γ production.

**Figure 3:**
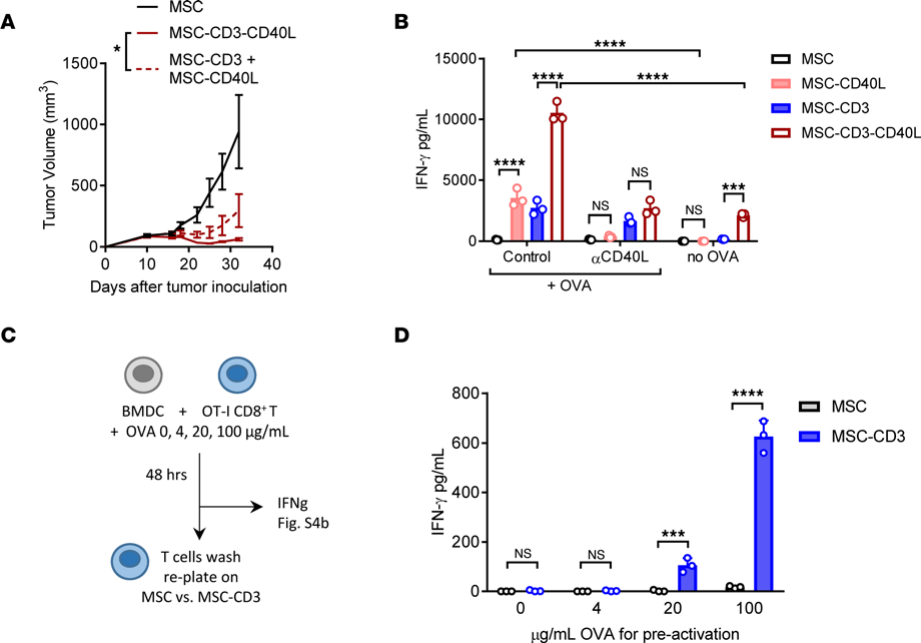
Exogenous TCR engager compliments endogenous antigen presentation for optimal T cell activation. (**A**) C57BL/6J mice were inoculated with 1 × 10^6^ MC38 tumor cells and treated peritumorally (p.t.) on the left (L) or right (R) side with 1 × 10^6^ MSC, MSC-CD3-CD40L (both L) or MSC-CD3 (R), and MSC-CD40L (L) on days 11, 14, and 17. (**B**) MSC, BMDC, and OT-I T cells were cocultured at a 1:1:10 ratio with or without OVA protein or α-CD40L blocking antibody for 48 hours, and IFN-γ in the supernatant was measured by cytometric bead array (CBA). (**C**) Schematic of experimental design for results from **D** and Supplemental Figure 4B. BMDC (10,000) were cocultured with OT-I CD8^+^ T cells purified from mouse spleen (100,000) with increasing concentrations of OVA peptide (0, 4, 20, 100 μg/mL) for 48 hours. Supernatant from coculture wells was collected for Supplemental Figure 4B, and OT-I CD8^+^ T cells were pooled from triplicate wells, washed, and reseeded onto MSC or MSC-CD3 for 48 hours. (**D**) IFN-γ production after additional 48 hours of coculture. Data are presented as mean ± SEM from a representative experiment (*n* = 5 [**A**] and *n* = 3 [**B** and **D**]) of 2 independent experiments. Statistical analysis was performed using 2-way ANOVA (**A**, **B**, and **D**) with Holm-Šidák multiple-comparison test (**B** and **D**). ****P ≤ 0.0001, ***P ≤ 0.001, *P ≤ 0.05.

We wondered whether the antitumor effects of TCR ligation and CD40 stimulation required spatial interactions or whether they were only additive benefits stemming from separate pathways. To test this, we injected MSC-CD3 on the left side of the tumor and MSC-CD40L on the right side of the tumor, and we compared antitumor efficacy with MSC-CD3-CD40L. Separating CD40L- and anti-CD3–expressing MSC on either side of the tumors led to decreased efficacy compared with treatment with MSC-CD3-CD40L in which both signals act in the same space (Figure 3A). While MC38 has abundant TIL, the host immune system fails to control tumors. We hypothesize that antigen presentation inside the TME is not optimized for full T cell activation. If that is the case, exogenous CD3 ligation and CD40 signaling can complement/boost tumor-reactive T cell reactivation in the presence of tumor antigen.

To better track T cell responses to antigens, we used OVA as a representative antigen. We used an in vitro coculture system in which MSC were seeded on the bottom of a 96-well plate, and BM-derived DCs (BMDC) loaded with OVA protein and purified CD8^+^ splenic T cells from OT-I mice were layered on top and allowed to interact. T cell activation was assessed by IFN-γ protein level in culture supernatants after 48 hours. We observed that anti-CD3 delivered by MSC can contribute to optimal antigen-specific responses when antigen is also presented, and it can further synergize with additional CD40L (Figure 3B). Therefore, both MSC-CD3 and MSC-40L significantly increase IFN-γ production from T cells being activated by a model tumor antigen (Figure 3B). The increase in IFN-γ seen by either MSC-CD40L or MSC-CD3-CD40L was returned to baseline after anti-CD40L blocking (Figure 3B). Furthermore, the level of IFN-γ produced by antigen-specific T cells was significantly greater than that produced after coculture with MSC-CD3-CD40L in an antigen-nonspecific context (Figure 3B). To further define T cell activation by antigen presentation from DC and additional CD3/TCR cross-linking, we assayed T cell activation using anti-CD3–coated plates and OVA-loaded BMDC. Without OVA, low-dose anti-CD3 coating did not increase IFN-γ production (Supplemental Figure 4A). However, during antigen-specific interactions with OVA access, further TCR cross-linking by anti-CD3 coating significantly increased IFN-γ production by antigen-specific T cells (Supplemental Figure 4A). T cells in the TME are already activated and have access to antigen prior to MSC therapy. Therefore, we sought to further characterize the effects of MSC activation on primed T cells. To do this, we preactivated OT-I CD8^+^ T cells with BMDC loaded with increasing concentrations of OVA protein for 48 hours (Figure 3C). During preactivation, antigen-specific T cells interacted with DCs presenting their cognate antigen and produced increasing levels of IFN-γ with increasing antigen (Supplemental Figure 4B). OT-I T cells from these wells were then washed and replated onto MSC to determine if they could be further activated after prior activation and IFN-γ production. With no or low antigen-specific preactivation, MSC-CD3 could not increase IFN-γ production by OT-I T cells (Figure 3D). However, with increasing antigen preactivation, subsequent activation by MSC-CD3 could further increase T cell activation (Figure 3D). This suggests that both further TCR cross-linking and activation of DCs by CD40L can optimize antigen-specific T cell activation.

### MSC-CD3-CD40L activates preexisting TILs and reshapes the TME.

**Figure 4:**
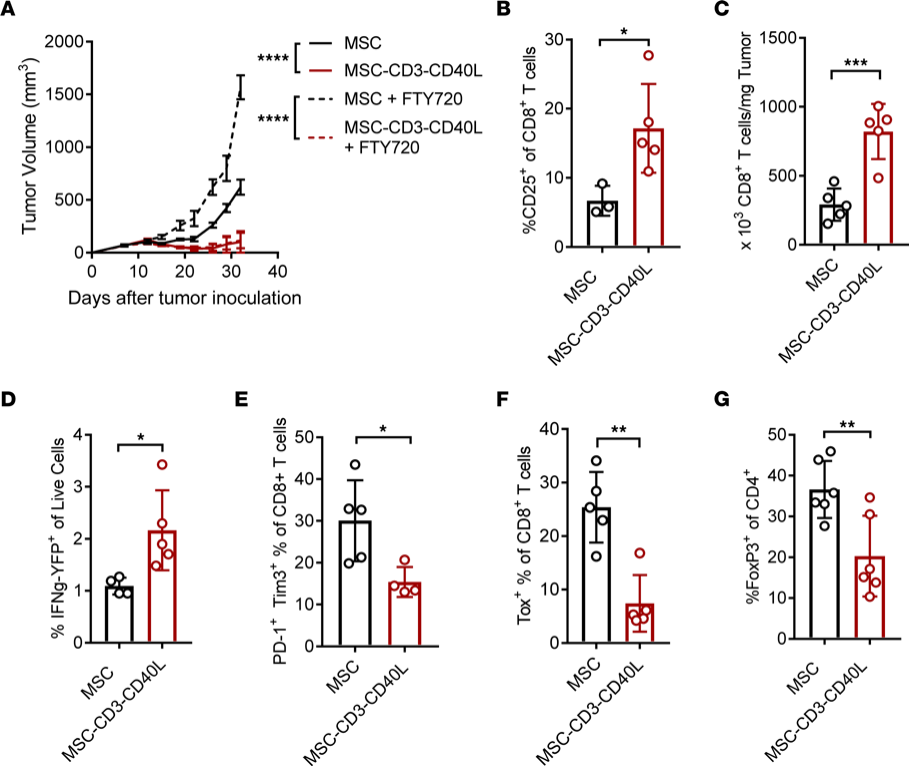
Combination therapy requires preexisting T cell immunity and reshapes the infiltrating T cell microenvironment. (**A**) C57BL/6J mice were inoculated with 1 × 10^6^ MC38 tumor cells and treated peritumorally (p.t.) with 1 × 10^6^ MSC or MSC-CD3-CD40L on days 11, 14, and 17 and treated with FTY720 20 μg 1 day before treatment; then, they were treated with 10 μg/mouse every 2 days for 3 weeks. (**B**–**G**) C57BL/6J mice were inoculated with 1 × 10^6^ MC38 tumor cells and treated (p.t.) with 1 × 10^6^ MSC or MSC-CD3-CD40L on days 11, 14, and 17. Tumors were removed 1 day after the first treatment (**B**) or 8 days after the first treatment (**C**–**G**) isolated into a single-cell suspension and analyzed by flow cytometry. Data are presented as mean ± SEM from a representative experiment (*n* = 3–5) of 2 independent experiments. Statistical analysis was performed using 2-way ANOVA (**A**) and 2-tailed unpaired Student’s *t* test (**B**–**G**). ****P ≤ 0.0001, ***P ≤ 0.001, **P ≤ 0.01, *P ≤ 0.05.

We next sought to determine where these cell interactions are occurring to produce tumor control. One possibility was that the MSC themselves were migrating to the draining lymph node (dLN) and were activating both T cells and DCs. However, this is unlikely because MSC were not detected in the dLN 1 day after therapy, but they were accumulated in the TME (Supplemental Figure 5). Other hypotheses included DCs being activated by MSC in the tumor and then traveling to the dLN to activate T cells, which then travel to the TME to control the tumor. Alternatively, DCs that are activated in the tumor by MSC reactivate T cells inside the TME, and activated T cells inside the TME can sufficiently control the tumor. To distinguish between these 2 possibilities, we used the S1P receptor agonist FTY720, which blocks T cells from leaving the dLN. If DCs migrate to the dLN to prime naive T cells, FTY720 will block their egress from the dLN, thereby blocking their antitumor effect. However if preexisting tumor-infiltrating DCs and T cells are primarily activated by engineered MSC, FTY720 treatment will have no impact on antitumor efficacy of MSC-CD3-CD40L. Using FTY720 to block newly primed T cell infiltration did not decrease the efficacy of MSC-CD3-CD40L (Figure 4A). Additionally, we observed the coexistence of MSC with DCs and CD8^+^ T cells in the same tumor sample by using IHC staining (Supplemental Figure 6, A and B). In MC38 tumors not injected with MSC, the stroma surrounding the tumor contained few cells and no colocalization of CD11c and CD8 cells (Supplemental Figure 6C). Taken together, these results suggest that the MSC-CD3-CD40L may generate exogenous TCR signaling inside tumors, leading to enhanced antitumor immunity.

Knowing that tumor-infiltrating CD8^+^ T cells were essential for therapeutic efficacy, we further dissected changes in the T cell compartment after treatment using flow cytometry. We saw an early activation of CD8^+^ T cells in the tumors of treated mice by increased CD25 expression 24 hours after the first treatment (Figure 4B). Two days after the last treatment, we saw an increase in CD8^+^ T cells (Figure 4C). To determine if there was an increase in functional CD8^+^ T cells in the TME after MSC-CD3-CD40L treatment, we used a transgenic IFN-γ reporter mouse that expresses yellow fluorescent protein (YFP) under the control of the endogenous IFN-γ promoter/enhancer regions. We inoculated MC38 tumors into IFN-γ–YFP transgenic mice and saw an increase in YFP^+^ CD8^+^ T cells in MSC-CD3-CD40L–treated tumors compared with controls 2 days after the last treatment (Figure 4D).

Recent work has also demonstrated the importance of T cell exhaustion in antitumor immunity (27–29). To assess the impact of MSC-CD3-CD40L on the exhausted state of tumor-infiltrating CD8^+^ T cells, we assessed PD-1^hi^Tim3^+^CD8^+^ T cells and measured TOX expression, both well-described markers of T cell exhaustion. Both PD-1^hi^Tim3^+^ and TOX^+^ CD8^+^ T cells were decreased in the TME after MSC-CD3-CD40L therapy (Figure 4, E and F). In the CD4^+^ T cell compartment, we saw a decrease in FoxP3^+^ cells of CD4^+^ T cells after MSC-CD3-CD40L treatment (Figure 4G). Therefore, MSC-CD3-CD40L works locally to reshape the TME by increasing functional CD8^+^ TILs and decreasing immunosuppressive Tregs.

### IL-12 production is critical for CD40 and T cell–stimulating therapy.

**Figure 5:**
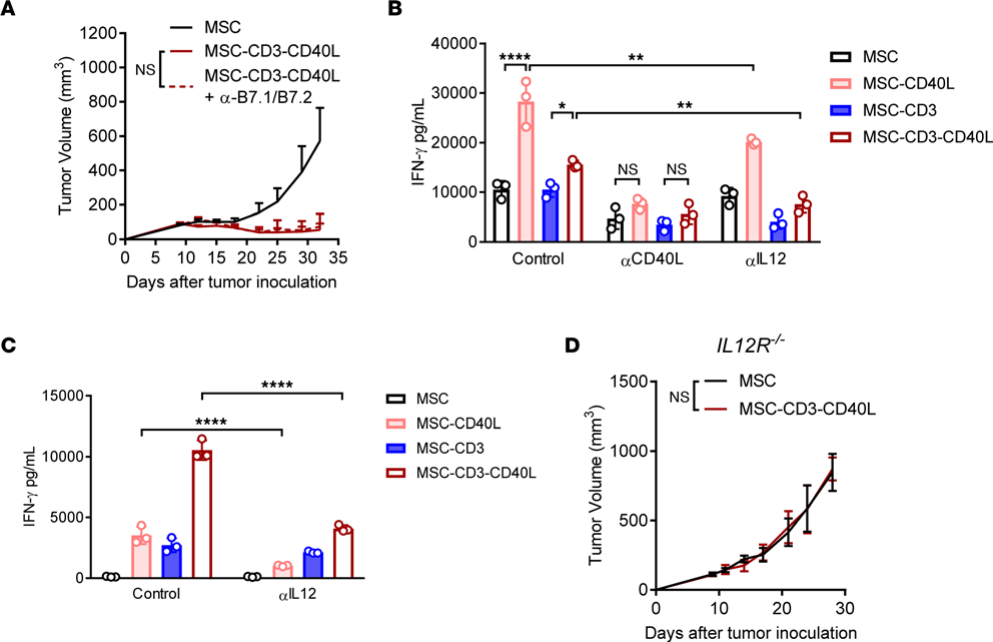
IL-12 is required for CD40-mediated T cell immunity. (**A**) C57BL/6J mice were inoculated with 1 × 10^6^ MC38 tumor cells and treated peritumorally (p.t.) with 1 × 10^6^ MSC or MSC-CD3-CD40L on days 11, 14, and 17 and treated with 200 μg each αB7.1/αB7.2 antibody twice weekly starting 1 day before treatment. (**B**) MSC were cocultured with BMDC at a 1:5 ratio for 24 hours with addition of α-CD40L or α–IL-12. Supernatant from MSC-BMDC coculture was transferred 1:1 into wells of OT-I T cells preactivated by α-CD3 coating for 24 hours. IFN-γ in the supernatant was measured 24 hours after supernatant transfer. (**C**) MSC, BMDC, and OT-I T cells were cocultured at a 1:1:10 ratio with OVA protein and α–IL-12 blocking antibody for 48 hours, and IFN-γ in the supernatant was measured by cytometric bead array (CBA). (**D**) C57BL/6J IL-12Rβ^–/–^ mice were inoculated with 1 × 10^6^ MC38 tumor cells and treated peritumorally (p.t.) with 1 × 10^6^ MSC or MSC-CD3-CD40L on days 11, 14, and 17. Data are presented as mean ± SEM from a representative experiment (*n* = 5 [**A** and **D**] and *n* = 3 [**B** and **C**]) of 2–3 independent experiments. Statistical analysis was performed using 2-way ANOVA (**A**–**D**) with Holm-Šidák multiple comparisons test (**B** and **C**). ****P ≤ 0.0001, **P ≤ 0.01, *P ≤ 0.05.

Knowing that both CD8^+^ T cells and DCs are required for antitumor efficacy of MSC-CD3-CD40L, we next sought to further explore if CD40 signaling on DCs led to upregulation of soluble and membrane mediators of T cell activation and, if so, to determine which molecules are essential for T cell activation. We cocultured BMDC and MSC expressing anti-CD3 and/or CD40L and analyzed CD80 expression as a marker of DC activation. CD80 was upregulated by MSC-CD40L and was blocked by anti-CD40L (Supplemental Figure 7A). However, in vivo blocking of both B7.1 and B7.2 (CD80 and CD86, respectively) did not abrogate the antitumor efficacy of MSC-CD3-CD40L (Figure 5A). MHCI was also upregulated on BMDC after coculture with MSC expressing CD40L, and it was blocked by anti-CD40L, suggesting that increased antigen presentation may also result from CD40 activation of DCs (Supplemental Figure 7B).

In addition to antigen presentation, MSC-activated DCs might also coordinately produce cytokines to further activate T cells. To determine whether a soluble mediator of DC activation could further activate T cells, we cocultured MSC and BMDC for 24 hours and then transferred the supernatant to OT-I T cells that had been stimulated in vitro for 24 hours by plate-bound anti-CD3. T cell activation after supernatant transfer was assessed by IFN-γ production after an additional 24 hours. We saw a significant increase in IFN-γ production by T cells cultured with supernatant from MSC-CD40L + BMDC cocultures and MSC-CD3-CD40L + BMDC cocultures, and this increase was CD40L dependent, as it was blocked by anti-CD40L (Figure 5B). Since T cells can be activated by IL-12 to produce IFN-γ, we determined if IL-12 from DCs is required for T cell activation and IFN-γ production. The IFN-γ production by T cells was significantly reduced when anti–IL-12 blocking antibody was added to MSC-BMDC cocultures prior to supernatant transfer (Figure 5B). IFN-γ signaling on DCs can also increase DC production of IL-12 independently of DC activation by CD40L (8). To determine the impact of IL-12–mediated T cell activation when MSC and BMDC are allowed to directly contact T cells, we blocked IL-12 signaling by anti–IL-12 during triple coculture of OT-I T cells, MSC, and BMDC + OVA, as described in Figure 3B. IFN-γ production was significantly decreased by blocking IL-12 in both the MSC-CD40L coculture and MSC-CD3-CD40L (Figure 5C). To confirm the role of IL-12 signaling in vivo, we inoculated IL12Rβ^–/–^ mice with MC38, and we treated them with control MSC or MSC-CD3-CD40L. There was no difference in tumor growth between treated and untreated mice (Figure 5D). These data suggest that IL-12 produced by MSC-activated DCs is essential for sufficient T cell activation and antitumor control.

### Local treatment induces systemic immune responses to control metastatic disease.

**Figure 6:**
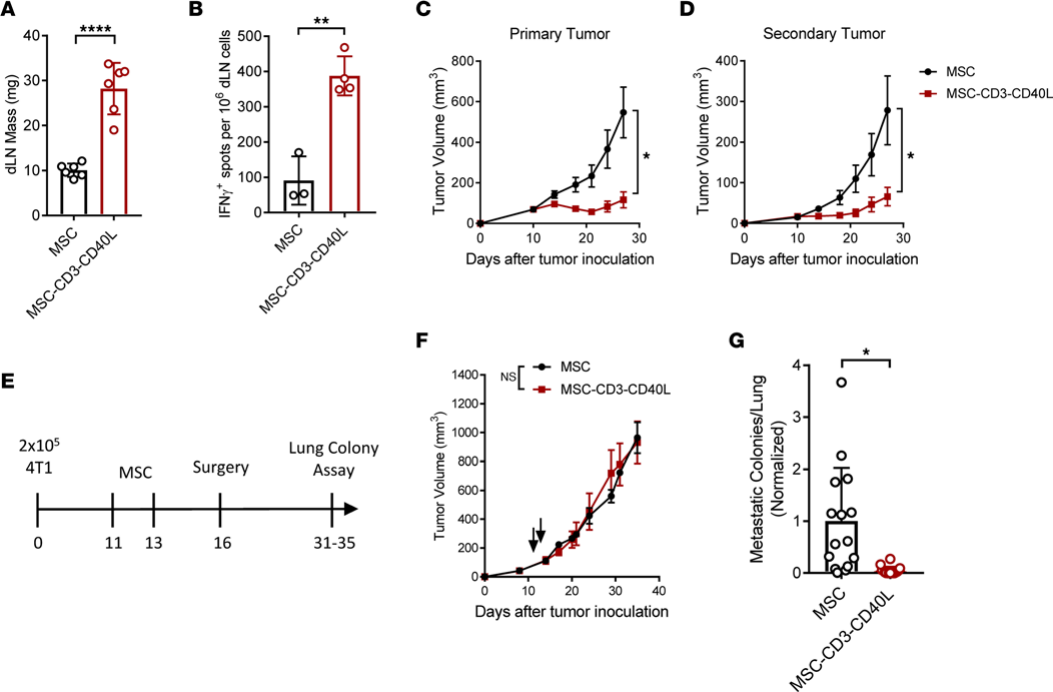
Exogenous first and second signals at local tumors control metastatic disease. (**A**) C57BL/6J mice were inoculated with 1 × 10^6^ MC38 tumor cells and treated peritumorally (p.t.) with 1 × 10^6^ MSC or MSC-CD3-CD40L on days 11, 14, and 17. Two days after the final treatment, the mass of dLN was measured. (**B**) C57BL/6J mice were inoculated with 1 × 10^6^ MC38 tumor cells and treated as described in **A**. Two days after the final treatment, the dLN was isolated to single cells and stimulated with irradiated MC38 tumor cells. Antigen-specific T cells were detected by ELISPOT assay and quantified. (**C** and **D**) C57BL/6 mice were inoculated on the left and right flank with MC38 and treated p.t. with 1 × 10^6^ MSC or MSC-CD3-CD40L on days 11, 14, and 17 on the primary tumor only. Tumor growth curves are shown. (**E**–**G**) BALB/c mice were inoculated with 2 × 10^5^ 4T1 tumor cells and treated p.t. with 1 × 10^6^ MSC or MSC-CD3-CD40L on days 11 and 13. Tumors were surgically removed on day 16, and lungs were removed for colony assay between days 31 and 35. Experimental design (**E**), tumor growth curves of mice without surgical removal of tumors (**F**), and normalized lung metastatic colonies (**G**) are shown. Data are presented as mean ± SEM from a representative experiment (*n* = 6 [**A**], *n* = 3–4 [**B**], and *n* = 5 [**C**, **D**, and **F**]) of 2–3 independent experiments. **G** represents 3 independent experiments with *n =* 4–5 pooled and normalized within experiment so MSC control–treated groups averaged to 1. Statistical analysis was performed using a 2-tailed *t* test (**A**, **B**, and **G**) or 2-way ANOVA (**C**, **D**, and **F**). ****P ≤ 0.0001, **P ≤ 0.01, *P ≤ 0.05.

One of the major goals of immunotherapy is to control metastatic disease. We next sought to determine if local T cell and DC activation by MSC-CD3-CD40L can generate sufficient systemic protection. We began by checking the dLN and observed that it is significantly enlarged 2 days after treatment compared with mice treated with control MSC (Figure 6A). More importantly, local MSC-CD3-CD40L treatment of the tumor led to significantly increased antigen-specific T cell responses in the dLN (Figure 6B). We next wanted to test the abscopal effects of MSC-CD3-CD40L treatment using a metastatic tumor model in which tumors were implanted on both the left and right flanks of mice, but only the primary tumor was treated with MSC-CD3-CD40L. Using the MC38 model, we saw not only a significant antitumor effect in the primary tumor, but also significantly decreased tumor growth in distal tumors (Figure 6, C and D). We further tested the abscopal response of MSC-CD3-CD40L in a spontaneous metastasis model using 4T1, a poorly immunogenic mammary tumor in the BALB/c background. In this tumor model, 4T1 cells were implanted on the flank and metastases begin to seed the lung during the second week. Tumors were treated with MSC on days 11 and 13; then, the primary tumor was surgically removed, as is often done in clinical practice. This allowed us to evaluate whether treatment can effectively control lung metastases seeded before surgery. Lungs were assayed for metastatic 4T1 growth between days 31 and 35 (Figure 6E). While MSC-CD3-CD40L had no effect on primary tumor growth (Figure 6F), it significantly reduced metastatic lung disease. In fact, many treated mice were metastasis free (Figure 6G). This suggests that, while MSC-CD3-CD40L only locally activates immune cells in the primary tumor, this activation can induce systemic antitumor immunity to treat metastatic diseases.

## Discussion

It has been challenging to address if and how tumor reactive T cells are reactivated either inside the TME or in dLN, as soluble drugs can flow into both tissues. We do know that low Ag/MHC complex expression can limit killing by cytotoxic T lymphocytes and that MHCI loss is correlated with poor survival in the clinic and resistance to checkpoint blockade (30–38). However, it is unclear whether T cell dysfunction inside the TME is attributable to lack of strong antigen engagement or costimulation. To explore if TCR engagement or costimulation is defective inside the TME and if exogenous TCR and costimulation could overcome these limitations, we carefully engineered autologous MSC capable of migrating into the TME and, once there, capable of providing either additional TCR simulation or costimulation to T cells. These cells can be used to determine if and which signals are limited for T cell activation and how to optimize T cell activation and overcome immunosuppression inside the TME. We show that local TCR cross-linking combined with CD40 activation reactivates preexisting TILs for efficient tumor control. Mechanistically, antitumor efficacy is dependent on CD8^+^ T cells and classical type I DCs (cDCs) in vivo. CD40 activation licenses DCs to promote CD8^+^ T cell immunity, while anti-CD3 can coordinately activate T cells that are partially activated by tumor cells. Taken together, our study highlights the important role of intratumoral DC licensing and MSC-DC CD8^+^ T cell cross-talk for optimum T cell activation inside the TME to maintain durable responses against cancer.

Early studies in mice showed that systemic T cell activation by administration of anti-CD3 leads to immediate cytokine release and associated toxicity, as well as immediate and long-lasting T cell dysfunction and depletion (39, 40). This toxicity is also seen in anti-CD3–based antibodies systemically administered to treat solid tumors (7). Simply providing exogenous CD3 signaling might not rescue dysfunctional TILs. We show that soluble anti–CD3-Fc in the TME actually depletes T cells, leading to increased tumor growth. Instead, membrane anti-CD3 is required for better T cell activation for tumor immunity. There is also a specific threshold of TCR stimulation required for T cell proliferation. Au-Yeung et al. show, using both in vitro and in vivo models, that T cells that commit to proliferation must pass a high threshold of TCR stimulation (41). This threshold for T cell activation is recalibrated by inhibitory receptor expression on T cells and cytokines in the TME (42, 43). Furthermore, Li et al. report that strong TCR signaling is positively correlated with memory T cell fate (44). Therefore, while strong TCR signaling can promote T cell proliferation and memory, if it is not delivered properly, it will eventually lead to T cell dysfunction. We therefore hypothesized that TCR engagement on T cells in the TME is not optimized. Interestingly, we describe that, even after coculture of CD8^+^ T cells with BMDC loaded with high concentrations of antigen, additional TCR ligation via MSC-CD3 cross-linking can increase IFN-γ production by antigen-specific T cells. Furthermore, the increase in IFN-γ production is most efficient in antigen-activated T cells, as MSC-CD3 do not robustly activate T cells without antigen. These in vitro and in vivo studies suggest that additional TCR signaling on antigen preactivated T cells in the TME can promote increased activation and function.

Several therapies target TCR/CD3 signaling in the TME, such as BiTEs and bispecific antibodies targeting CD3. While these therapies have shown promise in patients with residual hematopoietic malignancies, toxicity is still a major side effect for higher tumor load (45–47). Furthermore, while CD3-bispecific antibodies were shown to have immunostimulatory capabilities against solid tumors in preclinical models, they were unable to generate memory immune responses (48). Interestingly, acute lymphoblastic leukemia (ALL) blasts that were nonresponsive to BiTE therapy had higher surface PD-L1 expression, and blocking of CD80 signaling in preclinical ALL models led to decreased efficacy (49). The literature for BiTEs, therefore, suggests that CD3/TCR activation alone by tumor is insufficient for proper T cell activation and that costimulation may play an important role in efficacy. We show that, while MSC-CD3 signaling alone in the TME is not potent, addition of CD40L with spatiotemporal synchronicity leads to durable antitumor effects with memory formation. However, we also show that not all secondary signals increase antitumor efficacy when delivered concurrently with TCR engagement. MSC-CD3-CD80 delivered adjacent to the tumor have less antitumor effect compared with MSC-CD3 alone. CD80 has several binding partners, including CD28, PD-L1, and CTLA4 (50). One might speculate that CD80 directs MSC toward PD-L1–expressing tumor or suppressive myeloid cells, leading to reduced T cell activation. Additionally, CD80 on MSC may bind CTLA4 on T cells rather than CD28 and may interrupt functional TCR signaling. These results highlight the importance of secondary signals during TCR engagement, which are not normally present in bispecific anti-CD3–based therapies targeting nonhematopoietic solid malignancies.

In addition to increased TCR stimulation, MSC-CD3-CD40L provide APC activation to further optimize delivery of antigen to T cells in the TME. CD40L binding to APCs like DCs, macrophages, and B cells leads to induction of costimulatory molecules, cytokine production, and facilitation of cross-presentation of antigen (15). In the TME, it is known that CD40L plays a critical role in “DC licensing” and leads to optimum activation of CD8^+^ T cell immunity (17). We show that CD40L is decreased as tumors progress, suggesting that CD8^+^ T cell priming may be impaired over time. Supporting this hypothesis, we show that giving additional CD40L to supplement DC activation in the TME can synergize with TCR signaling for better antitumor control. The main pathway for CD40L/CD40 signaling in the TME is likely through myeloid cells; we and others have shown that tumor-infiltrating macrophages and DCs express CD40 receptor, but there is very little to no expression of CD40 receptor on CD8^+^ T cells (51). However Bourgeois et al. describe a role for CD40 expression on CD8^+^ T cells in memory cell fate (52). Therefore, while this is unlikely in our model, given the lack of expression of CD40 on CD8^+^ T cells, we cannot rule out a direct activation of CD8^+^ T cells by CD40L expression on MSC. Our data show a much clearer dependence on CD8^+^ T cells for tumor regression than CD4^+^ T cells. However, in our CD4^+^ depletion study, both Th cells and Tregs will be depleted. Theoretically, if an important expansion of Th subsets is balanced by Treg activity, total CD4^+^ T cell depletion will mask the Th effect. In our model, treated tumors have decreased Tregs in CD4^+^ T cells, suggesting a diminishing Treg-mediated immune suppression or an expansion of Th subsets. In this context, if Th subsets were critical for antitumor immunity, we would expect to see significant decreases in immunity when they are depleted. Taken together, we think these data show that, in our model, CD4^+^ T cells play a minor role in therapeutic efficacy compared with CD8^+^ T cells.

The importance of interactions between functional APCs and CD8^+^ T cells in the TME has not been fully explored. Recent preclinical and clinical studies have shown that stem-like and polyfunctional CD8^+^ T cells reside in APC niches and are critical predictors of patient survival (9, 53, 54). Preclinical studies have also confirmed that PD-L1 expression on DCs regulates the response to immune checkpoint blockade (55, 56). Furthermore, recent work shows that ovarian cancer patients who did not relapse after anti–PD-1 therapy were significantly enriched for an activated myeloid cell signature (53). Liu et al. also engaged CD3 signaling in the TME using an anti-CD3 × anti–PD-L1 fusion protein and show that therapy targets T cell activation proximal to uninhibited DCs, further supporting the notion that T cells can be optimally activated by coordination with DCs (7). Similarly, we found that targeting T cells to DCs by providing concomitant TCR stimulation and DC activation by MSC-CD3-CD40L also led to robust antigen-specific T cell activation requiring DCs. This activation requires proximity and is a further advantage of the ability to spatially and temporally control multiple-signal activation by MSC.

Most studies assessing CD40 agonist antibodies and targeting CD3^+^ T cell engager therapies systemically and have no way to confirm the role of dLN-derived T cells versus TILs. Furthermore, attempts to deliver CD40 agonists specifically into the TME by intratumoral injection led to significant antibody leaking into the dLN (57). Zhang et al. used TCR analysis of dLN and TILs, and they suggest that there may be migration of T cell from dLN to tumor after CD40 agonist therapy (51). However, without further interrogations, the direct effects of CD40 agonists on TILs is still unclear. We confirmed that local delivery of engineered MSC cannot directly impact dLN T cells, as MSC do not migrate out of the tumor. Instead, we confirm that CD40 and CD3 ligation are required in the tumor rather than the dLN using FTY720 and by spatially separating the CD40L and CD3 signals in the tumor. Interestingly, in the immune-resistant 4T1 model, MSC-CD3-CD40L failed to control the primary tumor but were able to decrease spontaneous lung metastases. MSC-CD3-CD40L rely primarily on intratumor T cells, which are scarce in 4T1 tumors. However, the microenvironment of 4T1 lung metastases is not well defined but may still support T cell–mediated antitumor responses generated from local tumor MSC-CD3-CD40L treatment. We demonstrate that, though local DC and T cell activation is transient, it generates systemic responses strong enough to control distal disease and durable enough to provide immunological memory.

Overall, our study demonstrates that T cell signaling in the TME can be further optimized by providing additional TCR cross-linking and DC costimulation. Tumor-targeted DC and T cell activation work synergistically to generate both local and systemic antitumor immune responses. This study is of general interest to those in the field of immunotherapy, oncology, and combination therapy.

## Methods

### Mice.

Six- to 8-week-old female C57BL/6J, BALB/c, C57BL/6J-Tg(TcraTcrb)1100Mjb/J (OT1 TCR transgenic), B6.129S4-*Ifng^tm3.1Lky^*/J (IFN-γ–YFP), B6(Cg)-Zbtb46^tm1(HBEGF)Mnz^/J (zDC-DTR), and B6.129S2-*Ighm^tm1Cgn^*/J (muMt^–^) mice were purchased from UT Southwestern breeding core or The Jackson Laboratory. All mice were maintained under specific pathogen–free conditions.

### Cell lines and reagents.

MC38, CT26, and 4T1 cell lines were purchased from ATCC. B16-OVA was a gift from Jinming Gao lab at UT Southwestern. All the cells were cultured at 37°C and 5% CO_2_ and were maintained in DMEM supplemented with 10% heat-inactivated FBS, 100 U/mL penicillin, and 100 μg/mL streptomycin and routinely tested for *Mycoplasma* contamination by PCR. Anti-CD8 (53-5.8), anti-CD4 (GK1.5), anti-CD40L (MR-1), anti–IL-12p75 (R2-9A5), anti-B7.1 (16-10A1), anti-B7.2 (GL-1), and anti–CSF-1Rβ (AFS98) were purchased from BioXCell. FTY720 was purchased from SelleckChem (Supplemental Table 1).

### Isolation, culture, and immortalization of BM-derived MSC.

BM-derived MSC were obtained according to previous methods, with a few modifications outlined briefly (58). BM cells were isolated from 6- to 8-week-old female C57BL/6J or BALB/c mice and plated in a single 10 cm tissue culture dish in MesenCult Expansion Media (Stemcell Technologies) with 1 μM GW2580 (Stemcell Technologies) to block the CSF-1 pathway in a chamber containing 1% oxygen. Media was changed every 3 days, and cells were passed 1:3 after reaching 60% confluence. MSC were identified by positive and negative surface marker expression (CD45^–^, CD11b^–^, Sca-1^+^, CD105^+^, CD44^+^). After passage 3 times, MSC were immortalized using retroviral transduction of SSR#69 vector, which was provided by T.C. He at the University of Chicago (Chicago, Illinois, USA).

### Generation of MSC-sCD3, MSC-CD3, MSC-CD40L, MSC-CD80, MSC-CD3-CD80, and MSC-CD3-CD40L cell lines.

293T cells were transfected with 3 plasmids (pLVX-sCD3-GFP, psPAX, and pMD2.G) to produce lentivirus encoding sCD3-GFP. Forty-eight hours later, supernatant containing lentivirus was harvested and filtered through 0.45 μm. Immortalized MSC were transduced with lentivirus. Two days after transduction, MSC were sorted by GFP expression. MSC-CD40L and MSC-CD80 were generated similarly, except that cells were sorted by CD40L and CD80 expression. MSC-CD3-CD40L and MSC-CD3-CD80 were generated by transducing MSC-CD3 with lentivirus encoding CD40L and CD80 and by sorting using surface marker expression equal to MSC-CD40L and MSC-CD80.

### Tumor growth and treatment.

MC38 (right flank 1 × 10^6^, left flank 5 × 10^5^), CT26 (5 × 10^5^), B16 (3 × 10^5^), and 4T1 (2 × 10^5^) cells were s.c. inoculated on only the right or both flanks of mice. After tumors grew to around 80–100 mm^3^, mice were randomly assigned to treatment groups. Mice were treated with 1 × 10^6^ MSC-GFP (MSC), MSC-sCD3 (secreting soluble anti–CD3-Fc), MSC-CD40L, 1 × 10^6^ MSC-CD3 (membrane-bound anti-CD3), or 1 × 10^6^ MSC-CD3-CD40L at the indicated time points. For CD8^+^ T cell depletion, CD4^+^ T cell depletion, CSF1Rβ-expressing cell depletion, and anti-B7.1/B7.2 blockade, 200 μg of antibodies was given intraperitoneally (i.p.) 1 day before the first treatment and twice weekly for 2–3 weeks. FTY720 (20 μg) was i.p. administered 1 day before treatment initiation, and then 10 μg was administered every other day for 2 weeks. DT was administered as a loading dose of 400 ng/mouse 1 day prior to therapy and maintained every other day at 200 ng/mouse. Tumor volmes were measured by the length, width, and height tumor volume was calculated by length × width × height/2. For the survival curve, mice were euthanized if each of length, width, or height of tumor is larger than 2 cm, the tumor volume exceeded 1,500 mm^3^ or if tumors had severe ulcerations requiring euthanasia.

### Tumor digestion.

Tumor tissues were excised and digested with 2.5 mg/mL Collagenase I (MilliporeSigma) and 0.5 mg/mL DNase I (Roche) at 37°C for 30–45 minutes. Digested tumor was then passed through a 70 μm cell strainer to remove large pieces of undigested tumor. Tumor-infiltrating cells were washed twice with PBS containing 2 mM EDTA and 2% FBS.

### Flow cytometry analysis.

Single-cell suspensions from tumor were incubated with anti-FcγIII/II receptor (clone 2.4G2) for 15 minutes to block nonspecific binding before staining with the conjugated antibodies or tetramer. 7-AAD Viability Staining Solution or Fixable Viability Dye eFluor 506 was used to exclude dead cells. Foxp3 and Tox were stained intracellularly using True-Nuclear transcription factor buffer set (BioLegend) following the manufacturer’s instructions. All staining steps were conducted at 4°C in the dark. Data were collected on CytoFLEX flow cytometer (Beckman Coulter) and analyzed by using CytExpert (Beckman Coulter) or FlowJo (Tree Star).

### IFN-γ ELISPOT.

MC38 tumor–bearing mice were treated with MSC, and 8 days after the first treatment, dLN cells were collected for single-cell suspension preparation. Cells were seeded in each well with irradiated MC38 tumor cells (3 × 10^4^) to stimulate the tumor-specific T cells. After 48 hours of culture, the ELISPOT assay was performed using the IFN-γ ELISPOT kit (BD Bioscience) according to the manufacturer’s instructions. IFN-γ spots were enumerated with the CTL-ImmunoSpot S6 Analyzer (Cellular Technology Limited).

### Generation of BM chimeras.

C57BL/6J mice were irradiated with 2 whole-body doses of 525 Rad given 3 hours apart. Irradiated mice were adoptively transferred (i.v.) with 3 × 10^6^ BM cells from zbtb46-DTR transgenic donor mice or WT mice within 12 hours. The mice were treated with sulfamethoxazole and trimethoprim antibiotics diluted in drinking water for 4 weeks after BM reconstitution. After 8 weeks, mice were inoculated with tumor cells.

### In vitro coculture of BMDC and T cells.

BMDC were generated from BM isolated from WT C57BL/6 mice cultured with 20 ng/mL recombinant mouse GM-CSF (BioLegend) in RPMI with 10% FBS for 7 days and were then counted and used in coculture experiments. CD8^+^ OT-I T cells were isolated from OT-I mouse spleens using magnetic bead isolation using EasySep Mouse CD8^+^ T cell Isolation Kit (Stemcell Technologies, 19853A) according to the manufacturer instructions. All coculture experiments were done in 96-well plates, and IFN-γ was measured in the supernatant after 48 hours by cytometric bead array using the Mouse Inflammation Kit (BD Biosciences, 552364). All samples received one-fifth the recommended reagents, as did the standard. In all experiments, CD40L was blocked at 50 μg/mL and IL-12p75 was blocked using 10 μg/mL. For triple coculture experiments, 1 × 10^4^ MSC were seeded with 1 × 10^4^ BMDC and 1 × 10^5^ OT-I CD8^+^ T cells, with varying concentrations of OVA protein, cultured for 48 hours; supernatant was collected. For BMDC experiments, 0.5 × 10^5^ BMDC were cocultured with 1 × 10^4^ MSC for 24 hours, and cells were trypsinized and stained with antibody and analyzed by flow cytometry. For supernatant transfer experiments, 1 × 10^5^ OT-I CD8^+^ T cells were seeded into wells coated with 1 μg/mL anti-CD3 (clone 17a2) for 24 hours; then, 100 μL of supernatant was removed and replaced with 100 μL of supernatant from wells containing 0.5 × 10^5^ BMDC that were cocultured with 1 × 10^4^ MSC for 24 hours. Detailed information for all antibodies is listed in Supplemental Table 1.

### IHC.

Formalin-fixed paraffin-embedded MC38 tumor slides were deparaffinized and rehydrated in xylene and gradient ethanol. First, antigen retrieval was performed in tris-based antigen retrieval buffer (Vector Laboratories) at 110°C for 8 minutes, followed by a wash with deionized H_2_0 (diH_2_O) and 1× PBS. Tissue sections were blocked with 10% goat serum (Vector Laboratories) for 1 hour, followed by incubation with primary antibody overnight SV40 (1:250, Abcam) at 4°C. Slides were washed with 1× PBS and incubated with alkaline phosphatase–conjugated (AP-conjugated) secondary anti–mouse antibody (catalog MP-5402 Vector Laboratories) for 30 minutes. Slides were then washed and developed with Warp Red Chromogen Kit (BioCare Medical). Multiplex staining was performed by stripping the previous antibody in citric-based antigen retrieval buffer (Vector Laboratories) at 110°C for 8 minutes before blocking and probing with the next primary antibodies: CD11c (1:100, Cell Signaling Technology) and CD8a (1:600, Cell Signaling Technology). Following similar steps from the first round of staining, slides were then incubated with HRP-conjugated secondary anti-rabbit antibody (Vector Laboratories) and developed with DAB (BioCare Medical). Slides were counter-stained with hematoxylin (Leica). Slides were scanned at 40× using the Hamamatsu Nanozoomer 2.0HT (Whole Brain Microscopy Facility, UT Southwestern).

### 4T1 metastatic tumor clonogenic assay.

4T1-bearing mice were p.t. treated with MSC or MSC-CD3-CD40L on days 11 and 13. Primary tumors were resected on days 16–18. Between 30 and 35 days after primary tumor inoculation, lungs were removed and digested with 1 mg/mL 488 Collagenase I (MilliporeSigma) and 0.5 mg/mL DNase I (Roche) at 37°C for 45 minutes. The digested cells were then passed through a 70 μm cell strainer for single-cell suspension. The resulting cells were resuspended and plated in DMEM supplemented with 10% heat-inactivated FBS, 100 U/mL penicillin, 100 μg/mL streptomycin, and 10 μg/mL thioguanine for clonogenic growth. After 4 days, cells were fixed with methanol and stained with crystal violet staining solution for counting colonies.

### Single-cell RNA-Seq data analysis.

Data for single-cell RNA-Seq analysis has been previously published (59). We compared CD40L expression among CD4^+^ T cells in dLN, spleen, and tumors from day 10 and day 20 to produce a heatmap of relative gene expression.

### Statistics.

All the experimental data analyses were performed with GraphPad Prism statistical software and shown as mean ± SEM. *P* value was determined by 2-way ANOVA for tumor growth, log-rank test for survival, and 2-tailed *t* tests or 1-way ANOVA for other analyses. When multiple comparisons were done, significance was adjusted by Holm-Šidák method. *P <* 0.05 was considered statistically significant.

### Study approval.

Animal experiments were conducted according to guidelines set by the IACUC of the UT Southwestern Medical Center. We performed no human research.

Author contributions

Conceptualization was contributed by CM, YXF, and JQ. Methodology was contributed by CM, LL, JB, and HL. Investigation was contributed by CM, JB, and HL. Writing of the original daft was contributed by CM. Review and editing of the manuscript were contributed by CM, LL, JB, YXF, and JQ. Funding acquisition was contributed by YXF, JQ. Supervision was contributed by YXF and JQ. CM and JB are co–first authors. CM initiated this study and is therefore listed first.

## Supplementary Material

Supplemental data

## Version 1 09/08/2022

Electronic publication
