# Supplementary material for: Exogenous signaling repairs defective T cell signaling inside the tumor microenvironment for better immunity
Source: JCI Insight. 2022 Sep 8;7(17):e159479. doi: 10.1172/jci.insight.159479 (PMC9536281; doi:10.1172/jci.insight.159479)
Supplement: Supplemental data [file jciinsight-7-159479-s047.pdf]

S1

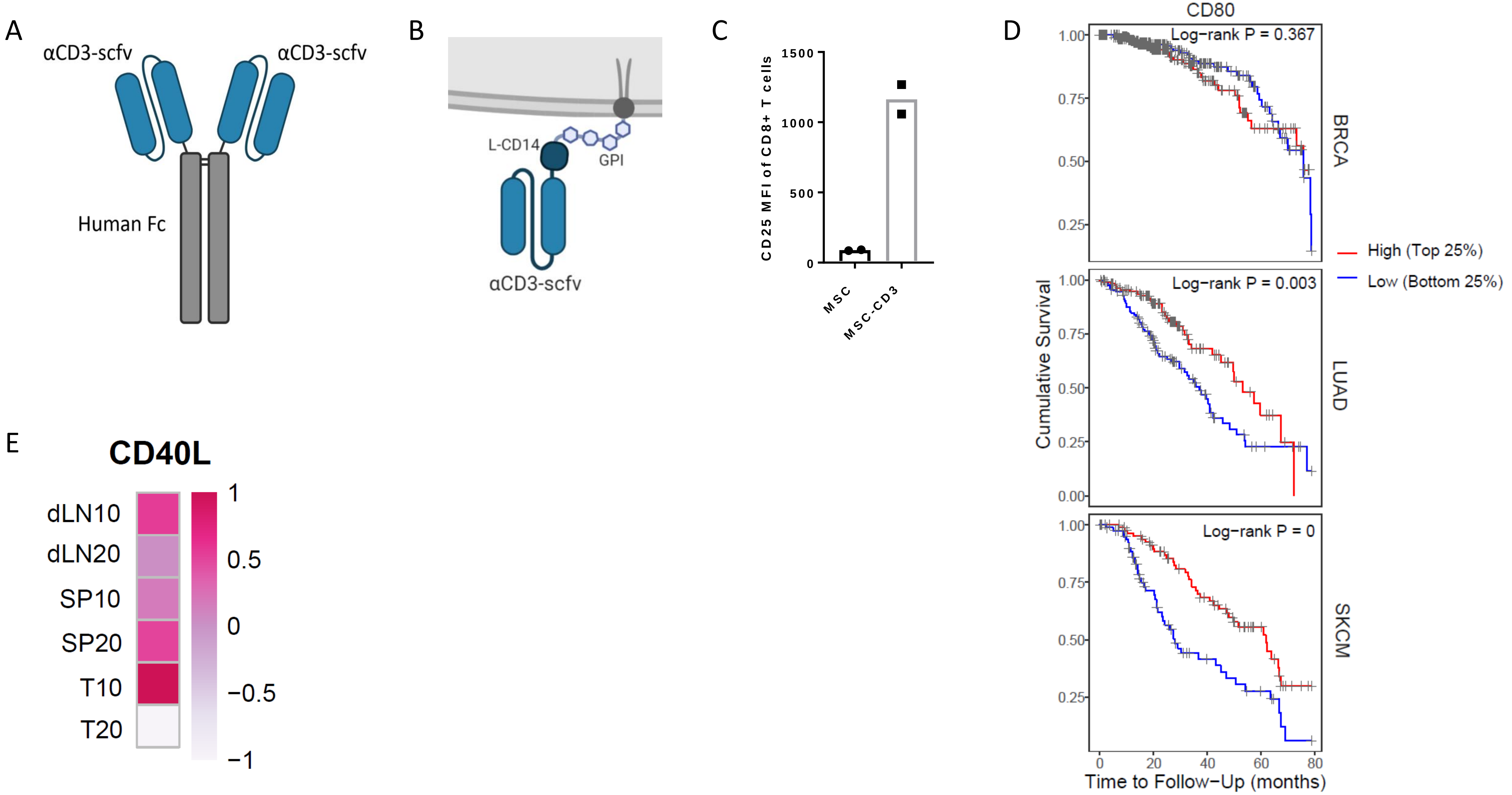

Supplementary Figure 1. Design of CD3 stimulation and impact of co-stimulation in the TME.
A Schematic of soluble αCD3 secreted by MSC-sCD3 in Figure 1A-B. **B** Schematic of membrane-bound αCD3 expressed by MSC-CD3 referenced in Figure 1D and throughout the manuscript. It is GPI anchored using the leading sequence of CD14. **C** CD25 expression on CD8+ T cells when co-cultured with MSC vs. MSC-CD3. **D** Cumulative survival in Breast Invasive Carcinoma (BRCA), Lung Adenocarcinoma (LUAD) and Skin Cutaneous Melanoma (SKCM) patients according to CD80 expression (top 25% vs. bottom 25%). **E** Relative expression of CD40 ligand on CD4+ T cells in draining lymph node (dLN) spleen (SP) and tumor (T) from day 10 and day 20 after subcutaneous MC38 tumor inoculation.

S2

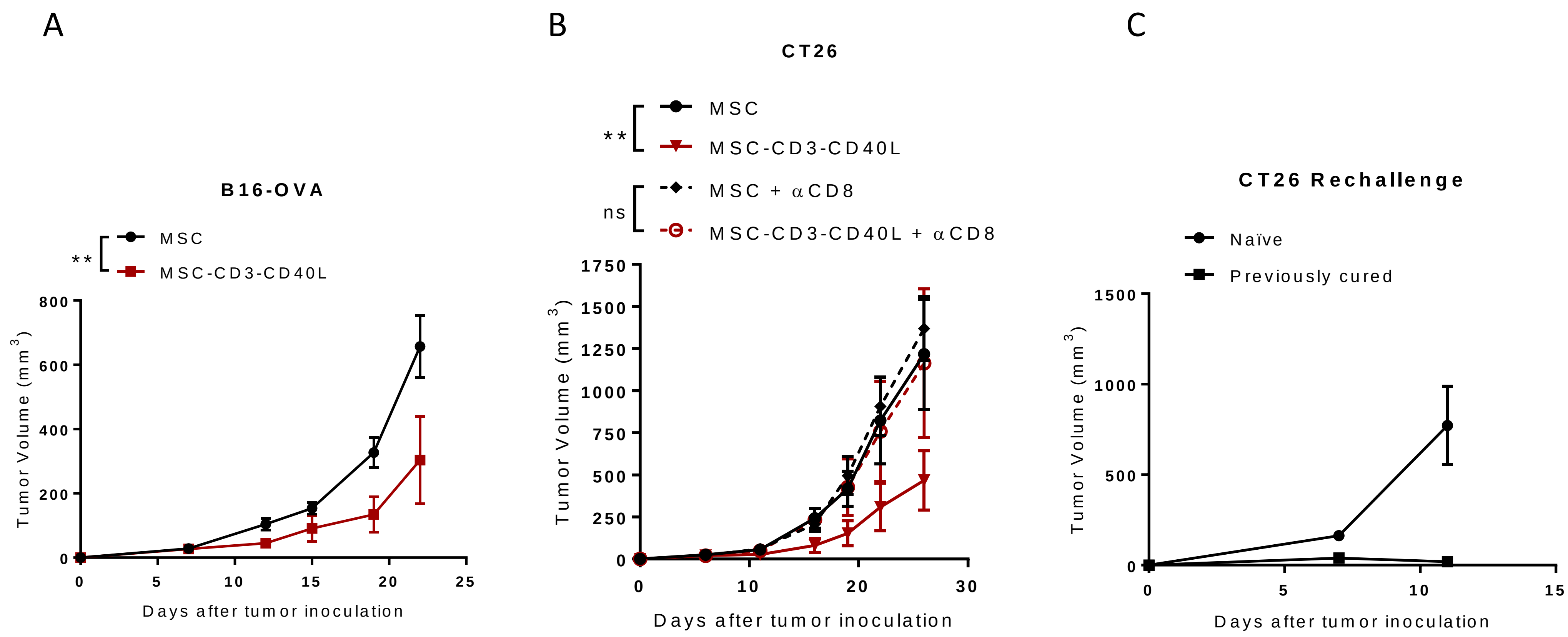

Supplementary Figure 2. Efficacy of MSC-CD3-CD40L in pre-clinical models.
A-B Balb/c mice were inoculated with $5x10^5$ CT26 tumor cells (**A**) or C56BL/6J mice were inoculated with $3x10^5$ B16-OVA tumor cells (**B**) and treated (p.t.) on days 8, 11, 14 with $1x10^6$ of MSC or MSC-CD3-CD40L. Tumor growth was measured twice per week. (**C**) Two months after being cured of CT26, Balb/c mice we re-challenged with 5 million CT26 tumor cells on the opposite flank and tumor growth was monitored compared to naïve Balb/c mice. Statistical analysis was performed using two-way ANOVA. **P ≤ 0.01, ns not significant.

S3

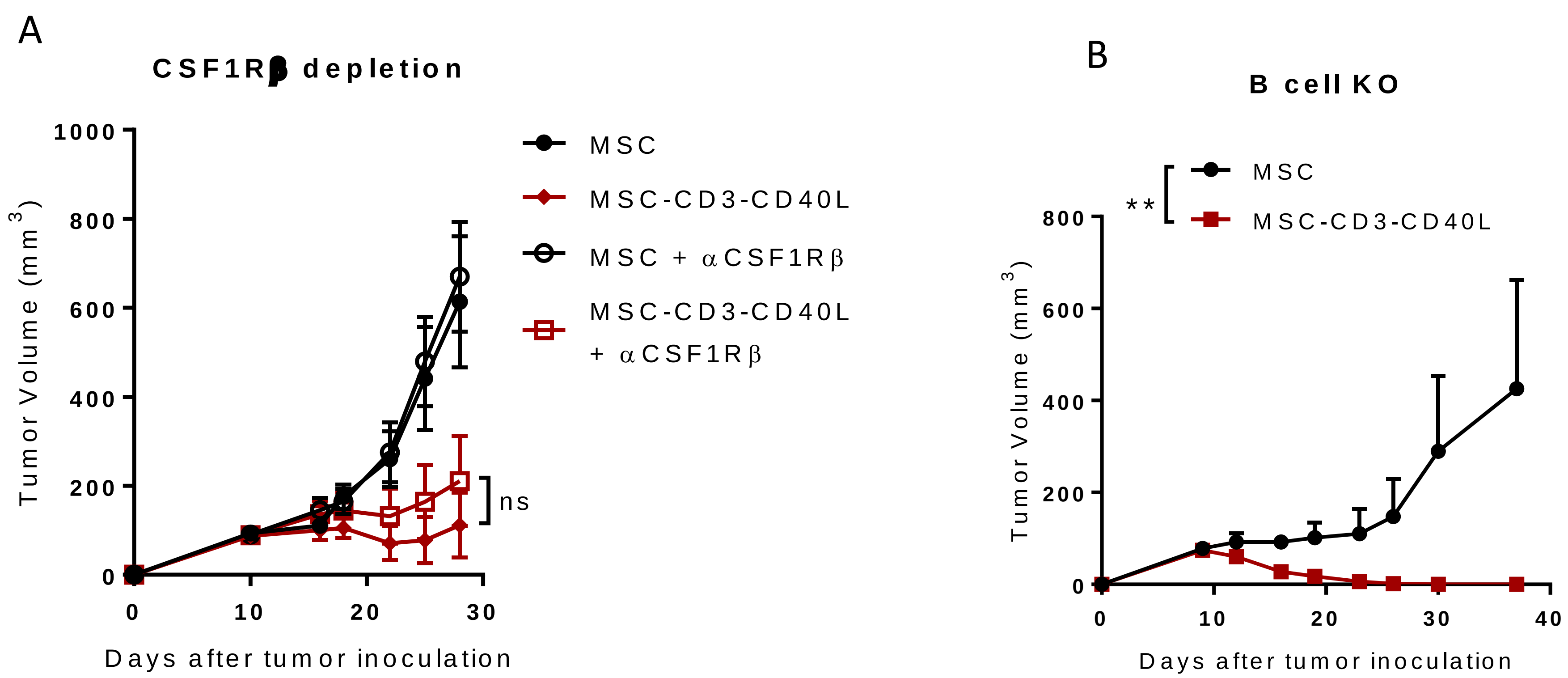

Supplementary Figure 3. Macrophage depletion does not impact MSC-CD3-CD40L efficacy.
A C56BL/6J mice were inoculated with $1x10^6$ MC38 tumor cells and treated (p.t.) on days 11, 14, 17 with $1x10^6$ of MSC or MSC-CD3-CD40L and macrophages were depleted using 200ug/dose α-CSF1Rβ beginning 1 day before MSC treatment and continuing twice per week for 2 weeks. **B** MC38 bearing $MuMt^-$ mature B cell deficient mice were treated with MSC or MSC-CD3-CD40L beginning on day 11. Tumor growth was measured twice per week. Data are presented as mean ± s.e.m. Statistical analysis was performed using two-way ANOVA. **P ≤ 0.01, ns not significant.

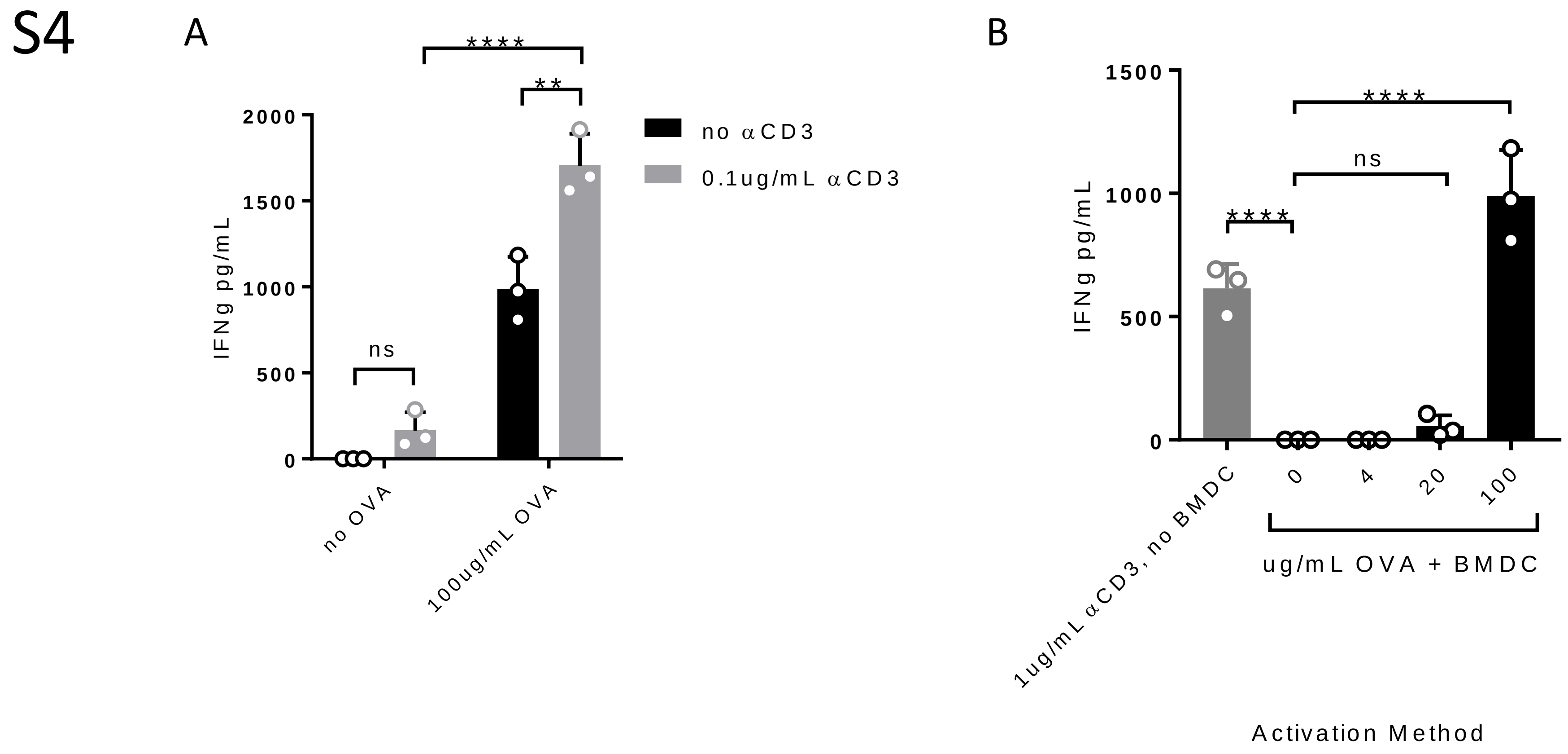

Supplementary Figure 4. Antigen specific T cell activation by BMDC priming vs. CD3 ligation.
A BMDC (10k) and splenic OT-I CD8+ T cells (100k) were co-cultured with or without 100ug/mL OVA protein on plates with or without pre-coating of 0.1ug/mL αCD3. After 48hrs of co-culture, IFNg in the supernatant was measured by CBA. **B** BMDC (10k) and splenic OT-I CD8+ T cells (100k) were co-cultured with 0, 4, 20 or 100ug/mL OVA protein (see Figure 3c for schematic). High dose 1ug/mL coated anti-CD3 is provided for perspective. After 48hrs co-culture supernatant IFNg was measured by CBA. Data are presented as mean ± SD from a representative experiment (n=3) of 2 independent experiments. Statistical analysis was performed using two-way ANOVA with Sidak's multiple comparisons test (A) and one-way ANOVA with Dunnett's multiple comparisons test (B). ****P ≤ 0.0001, **P ≤ 0.01, ns not significant.

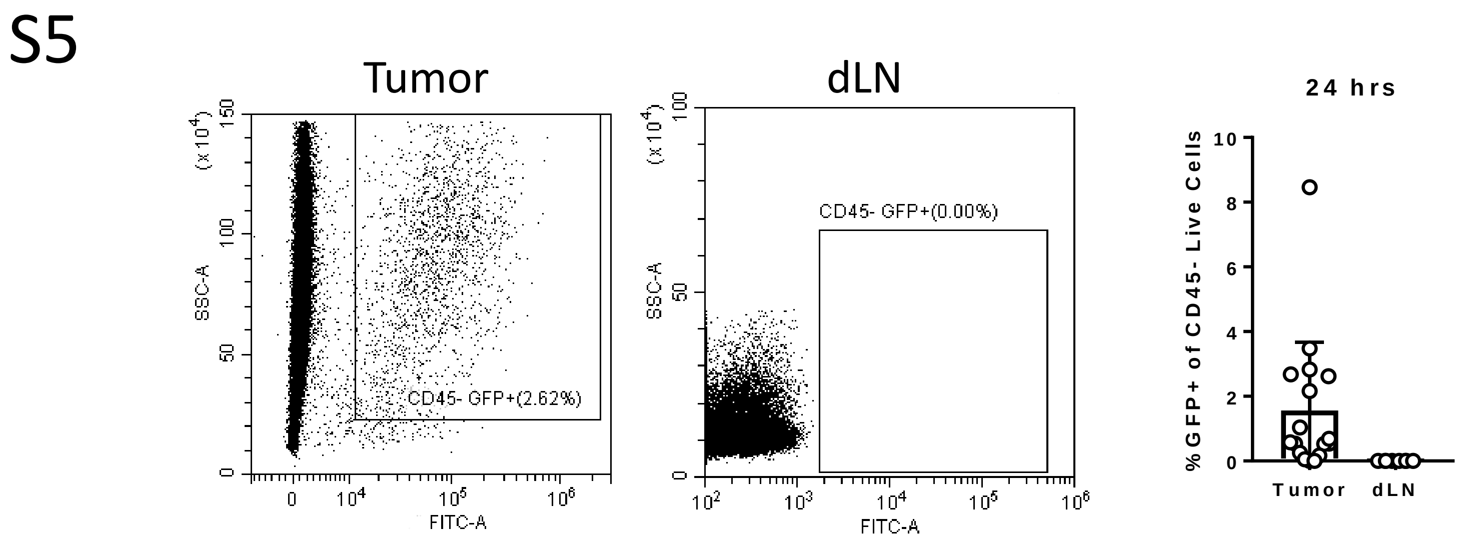

Supplementary Figure 5. MSC-CD3-CD40L migration into the TME vs. dLN.
C57BL/6J mice were inoculated with $1x10^6$ MC38 tumor cells and treated (p.t.) with $1x10^6$ MSC or MSC-CD3-CD40L on day 11. Tumors and draining lymph node (dLN) were removed 1 day after the first treatment, isolated into a single cell suspension and analyzed by flow cytometry. Left is representative flow cytometry plots showing GFP on X axis in Tumor vs. dLN of live cells. Right shows quantitation of CD45- GFP+ cell percentages.

S6

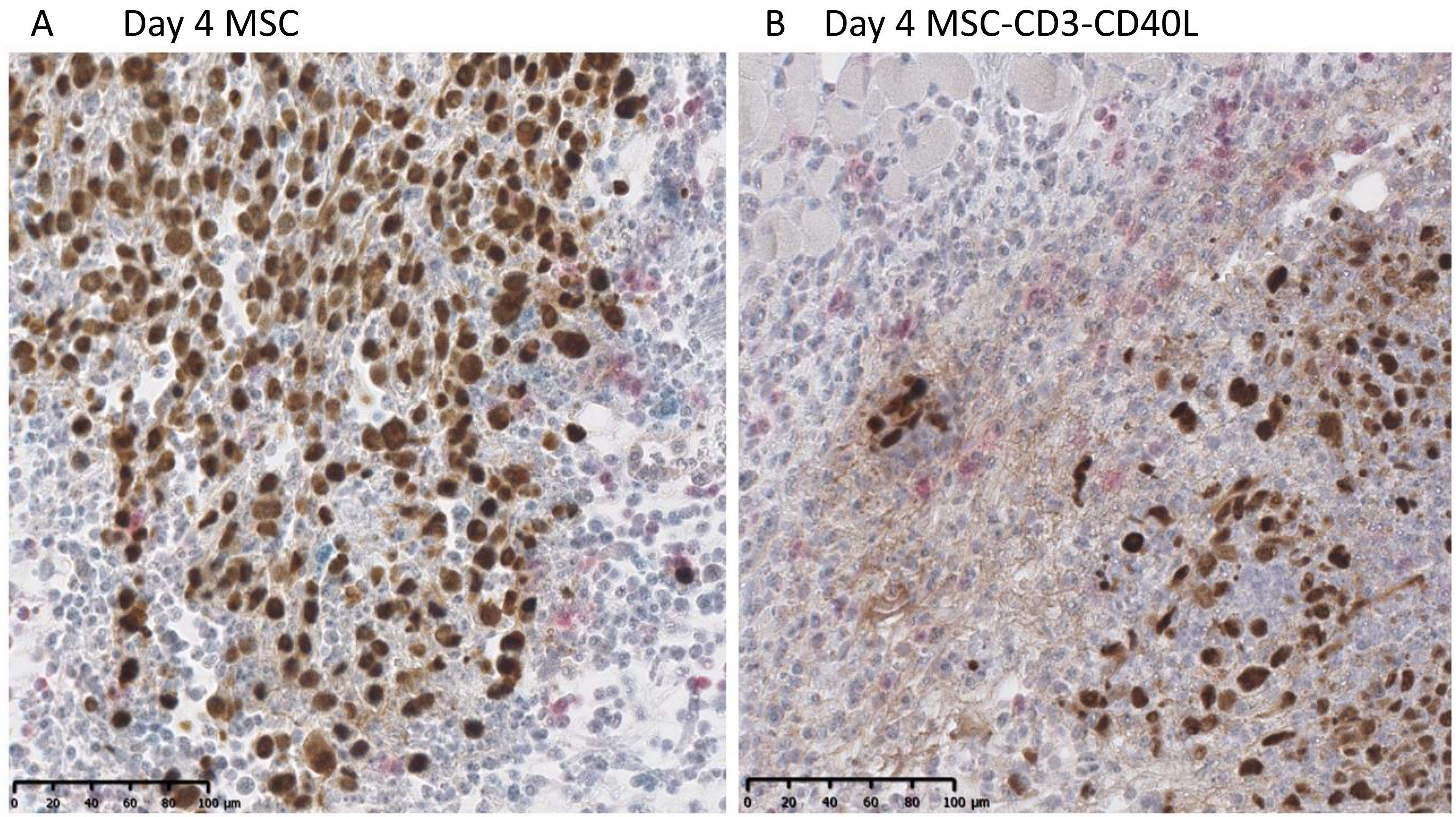

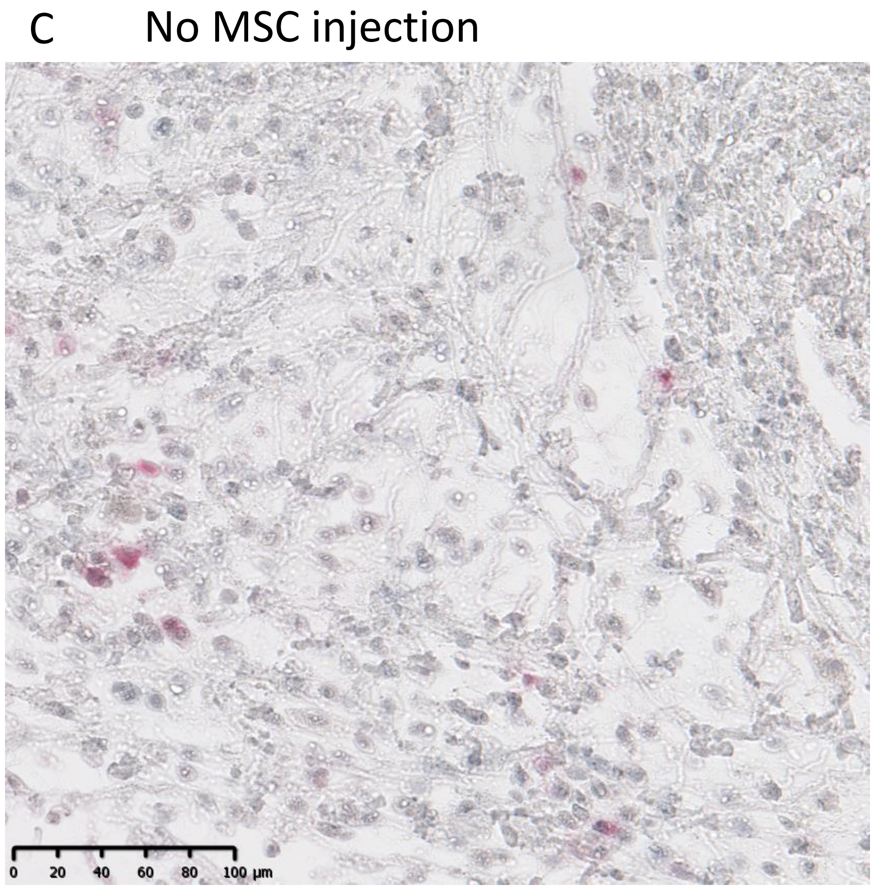

Supplementary Figure 6. Co-localization of MSC, CD8+ and CD11c+ cells in the TME.
A-C Representative images of formalin fixed and stained tumor collected 4 days after peri-tumor injection of MSC stained for SV40 (brown), CD8 (red) and CD11c (blue) treated with MSC (**A**), MSC-CD3-CD40L (**B**) or no MSC (**C**). Scale bar shown in lower left corner.

S7

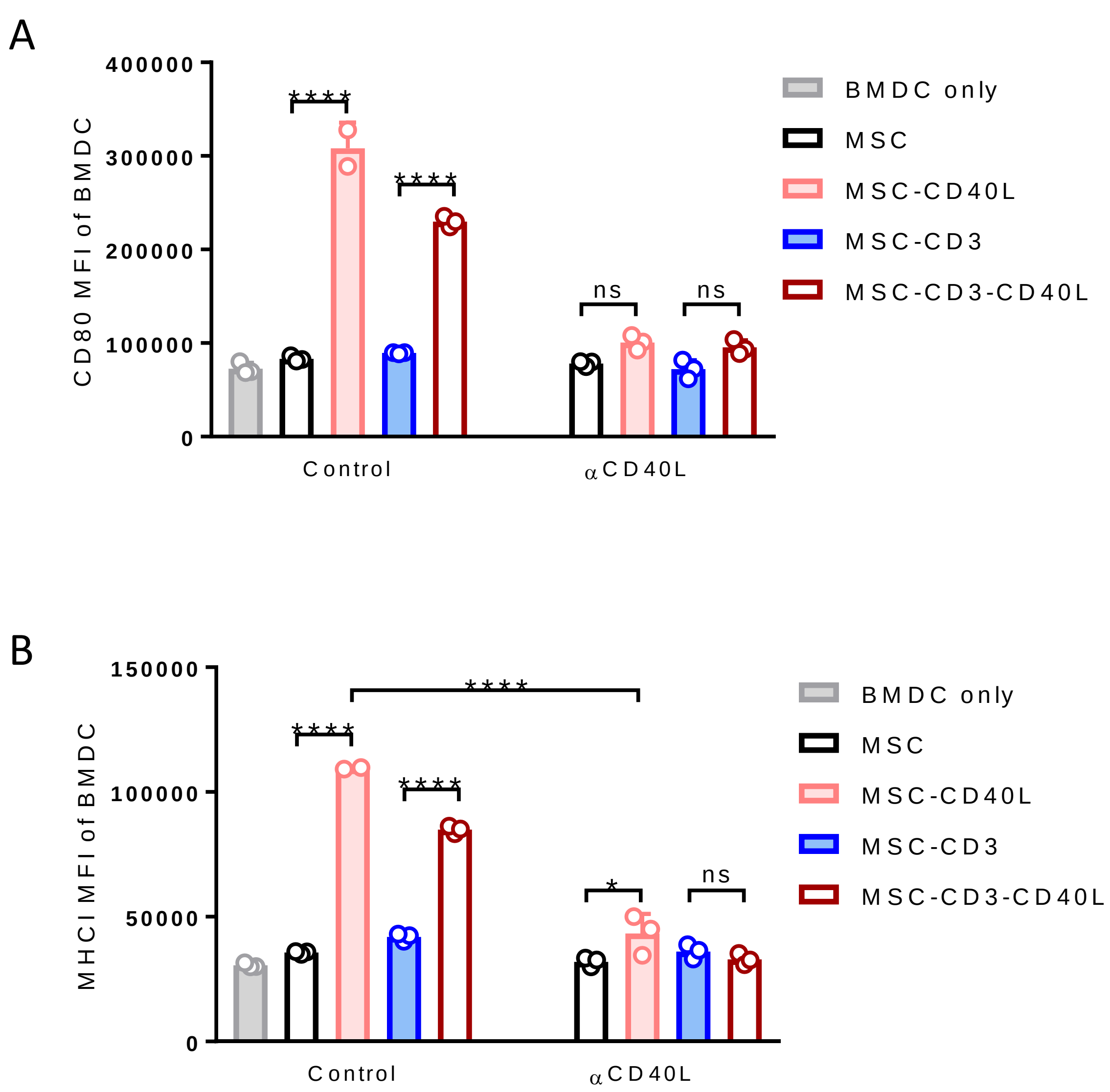

Supplementary Figure 7. MSC co-activator expression increases BMDC activation markers.
A BMDC (50k) and MSC (10k) were co-cultured for 24 hours with or without αCD40L blocking antibody. Cells were trypsinized, stained with antibodies then analyzed by flow cytometry for CD80 expression (**A**) and MHCI expression (**B**). Data are presented as mean ± SD from a representative experiment (n = 3) of 2 independent experiments. Statistical analysis was performed using two-way ANOVA (A) and two-tailed unpaired Student's t-test with Sidak's multiple comparisons test. ****P ≤ 0.0001, *P ≤ 0.05, ns not significant.

Supplemental Table 1. Key reagents table

Reagent or resource	Source	Identifier
Antibodies		
Anti–mouse CD45 (flow cytometry, 30-F11)	BioLegend	catalog 103126
Anti–mouse CD3 (flow cytometry, 145-2C11)	BD Biosciences	catalog 564379
Anti–mouse CD8 (flow cytometry, 53-6.7)	BioLegend	catalog 100730
Anti–mouse CD4 (flow cytometry, RM4-5)	BD Biosciences	catalog 550954
Anti–mouse PD-1 (flow cytometry, 29F.1A12)	BioLegend	catalog 135224
Anti–mouse TIM-3 (flow cytometry, RMT3-23)	eBioscience	catalog 25587008
Anti–mouse TOX (flow cytometry, TXRX10)	eBioscience	catalog 50-6502-82
Anti–mouse Foxp3 (flow cytometry, MF-14)	BioLegend	catalog 126408
Anti–mouse CD25 (flow cytometry, PC61)	BD Biosciences	catalog 564021
Anti–mouse CD11b (flow cytometry, M1/70)	eBioscience	catalog 12-0112-82
Anti–mouse CD11c (flow cytometry, HL3)	BD Biosciences	catalog 550261
Anti–mouse F4/80 (flow cytometry, BM8)	BioLegend	catalog 123116
Anti–mouse CD103 (flow cytometry, 2E7)	BioLegend	catalog 121406
Anti–mouse CD19 (flow cytometry, 6D5)	BioLegend	catalog 115508
Anti–mouse I-A/I-E (flow cytometry, M5.114.15.2)	eBioscience	catalog 56-5321-82
Anti–CD16/32 (clone 2.4G2)	In house	N/A
Purified anti-mouse CD3 (17A2)	BioLegend	catalog 100202
Fixable Viability Dye eFluor 780	Thermo Fisher Scientific	catalog 65-0866-18
H-2Kb MuLV p15E Tetramer-KSPWFTTL-PE	MBL	catalog TB-M507-1
7-AAD Viability Staining Solution (flow cytometry)	BioLegend	catalog 420404
InVivoMAb anti–mouse CSF-1Rβ (CD115) (AFS98)	BioXCell	catalog BE0213
InVivoMAb anti–mouse CD8 (53-5.8)	BioXCell	catalog BE0223
InVivoMAb anti–mouse CD4 (GK1.5)	BioXCell	catalog BE0003-1
InVivoMAb anti–mouse CD80 (B7-1) (1G10)	BioXCell	catalog BE0024
InVivoMAb anti–mouse CD86 (B7-2) (GL-1)	BioXCell	catalog BE0025
InVivoMAb anti–mouse CD40L (CD154) (MR-1)	BioXCell	catalog BE0017-1
InVivoMAb anti–mouse IL-12p75 (R29A5)	BioXCell	catalog BE0233
Bacterial and virus strains		
N/A		
Biological samples		
N/A		
Chemicals, peptides, and recombinant proteins		
FTY720 (hydrochloride)	SelleckChem	catalog S5002
DT from corynebacterium diphtheriae	MilliporeSigma	catalog D0564
DMEM	Sigma-Aldrich	catalog D6429
RPMI-1640	Sigma-Aldrich	catalog R8758-500ML
MesenCult Expansion Media	Stemcell Technologies	catalog 05513
Collagenase type I	MilliporeSigma	catalog C0130
OVALBUMIN ENDOFIT	Invivogen	catalog vac-pova
DNase I	Roche	catalog 11284932001
GW2580	Stemcell Technologies	catalog 72472

continued on next page

Reagent or resource	Source	Identifier
Critical commercial assays		
BD Mouse IFN-γ ELISPOT Sets	BD Biosciences	catalog 551083
True-Nuclear Transcription Factor Buffer Set	BioLegend	catalog 424401
CD45 (TIL) MicroBeads, mouse	Miltenyi Biotec	catalog 130-110-618
BD Cytometric Bead Array (CBA) Mouse Inflammation Cytokine Kit	BD Biosciences	catalog 560485
Deposited data		
N/A		
Experimental models: Cell lines		
MC38	ATCC	N/A
CT26	ATCC	catalog CRL-2683
B16-OVA	Gao Lab	N/A
4T1	ATCC	catalog CRL-2539
293T	ATCC	catalog CRL-3216
Experimental models: Organisms/strains		
C57BL/6J	The Jackson Laboratory	Strain 000664
B6.129S4-Ifng$^{tm3.1Lky}$/J	The Jackson Laboratory	Strain 017581
B6.129S(C)-Batf3^{tm1Kmm}/J	The Jackson Laboratory	Strain 013755
B6(Cg)-Zbtb46$^{tm1(HBEGF)Mnz}$/J	The Jackson Laboratory	Strain 019506
C57BL/6-Tg(TcraTcrb)1100Mjb/J	The Jackson Laboratory	Strain 003831
B6;129S1-Il12rb2^{tm1Jm}/J	The Jackson Laboratory	Strain 003248
B6.129S2-Ighm^{tm1Cgn}/J	The Jackson Laboratory	Strain 002288
Oligonucleotides		
N/A		
Recombinant DNA		
SSR#69 Immortalization Retroviral Vector	Gift from T.C. He	N/A
Plasmid: pMD2.G		
Plasmid: psPAX2		
Plasmid: pLVX-sCD3-GFP	This paper	N/A
Plasmid: pLVX-CD3-GFP	This paper	N/A
Plasmid: pLVX-CD40L-GFP	This paper	N/A
Plasmid: pLVX-CD80-GFP	This paper	N/A
Software and algorithms		
GraphPad Prism software 7.0	GraphPad Software Inc.	https://www.graphpad.com/scientific-software/prism/
CTL-ImmunoSpot S6 Analyzer	Cellular Technology Limited	https://immunospot.com/products/analyzers
CytExpert	Beckman Coulter	https://www.beckman.com/flow-cytometry/research-flow-cytometers/cytoflex/software
BD FACSChorus Software	BD Biosciences	https://www.bdbiosciences.com/en-us/products/software/instrument-software/bd-facschorus-software
FlowJo	Tree Star Inc.	https://www.flowjo.com/solutions/flowjo
TIMER	Ref. 24	https://cistrome.shinyapps.io/timer/
Other		
N/A		
